# *Marrubium alysson* L. Ameliorated Methotrexate-Induced Testicular Damage in Mice through Regulation of Apoptosis and miRNA-29a Expression: LC-MS/MS Metabolic Profiling

**DOI:** 10.3390/plants11172309

**Published:** 2022-09-03

**Authors:** Reda F. A. Abdelhameed, Asmaa I. Ali, Sameh S. Elhady, Hend E. Abo Mansour, Eman T. Mehanna, Sarah M. Mosaad, Salma A. Ibrahim, Rawan H. Hareeri, Jihan M. Badr, Nermeen A. Eltahawy

**Affiliations:** 1 Department of Pharmacognosy, Faculty of Pharmacy, Galala University, New Galala 43713, Egypt; 2 Department of Pharmacognosy, Faculty of Pharmacy, Suez Canal University, Ismailia 41522, Egypt; 3 Department of Pharmacognosy, Faculty of Pharmacy, Misr International University, Cairo 12585, Egypt; 4 Department of Natural Products, Faculty of Pharmacy, King Abdulaziz University, Jeddah 21589, Saudi Arabia; 5 Department of Biochemistry, Faculty of Pharmacy, Menoufia University, Shebin El-Koum 32511, Egypt; 6 Department of Biochemistry, Faculty of Pharmacy, Suez Canal University, Ismailia 41522, Egypt; 7 Division of Pharmacology and Therapeutics, Department of Continuous Medical Education, General Authority of Healthcare, Ismailia 41522, Egypt; 8 Department of Pharmacology and Toxicology, Faculty of Pharmacy, King Abdulaziz University, Jeddah 21589, Saudi Arabia

**Keywords:** sustainability of natural resources, drug discovery, *Marrubium alysson* L., metabolomic profiling, methotrexate, testicular damage, miRNA-29a, antioxidant, anti-inflammatory, antiapoptotic

## Abstract

Despite the efficient anti-cancer capabilities of methotrexate (MTX), it may induce myelosuppression, liver dysfunction and testicular toxicity. The purpose of this investigation was to determine whether *Marrubium alysson* L. (*M. alysson* L.) methanolic extract and its polyphenol fraction could protect mouse testicles from MTX-induced damage. We also investigated the protective effects of three selected pure flavonoid components of *M. alysson* L. extract. Mice were divided into seven groups (n = 8): (1) normal control, (2) MTX, (3) Methanolic extract + MTX, (4) Polyphenolic fraction + MTX, (5) Kaempferol + MTX, (6) Quercetin + MTX, and (7) Rutin + MTX. Pre-treatment of mice with the methanolic extract, the polyphenolic fraction of *M. alysson* L. and the selected pure compounds ameliorated the testicular histopathological damage and induced a significant increase in the serum testosterone level and testicular antioxidant enzymes along with a remarkable decline in the malondialdehyde (MDA) level *versus* MTX alone. Significant down-regulation of nuclear factor kappa B (NF-κB), tumor necrosis factor-alpha (TNF-α), p53 and miRNA-29a testicular expression was also observed in all the protected groups. Notably, the polyphenolic fraction of *M. alysson* L. displayed a more pronounced decline in the testicular levels of interleukin-1β (IL-1β), interleukin-6 (IL-6) and MDA, with higher testosterone levels relative to the methanolic extract. Further improvements in the Johnsen score, histopathological results and all biochemical assays were achieved by pre-treatment with the three selected pure compounds kaempferol, quercetin and rutin. In conclusion, *M. alysson* L. could protect against MTX-induced testicular injury by its antioxidant, anti-inflammatory, antiapoptotic activities and through the regulation of the miRNA-29a testicular expression. The present study also included chemical profiling of *M. alysson* L. extract, which was accomplished by LC-ESI-TOF-MS/MS analysis. Forty compounds were provisionally assigned, comprising twenty compounds discovered in the positive mode and seventeen detected in the negative mode.

## 1. Introduction

Implementation of chemotherapy served in the improvement of the survival rates in many cases of malignanciy. However, the use of chemotherapy is accompanied by organ toxicity and intense oxidative injury [1]. Methotrexate (MTX) is a folate antagonist drug that is widely used for the treatment of various autoimmune diseases and some cancer types. MTX mediates its anticancer effect through the induction of oxidative stress as well as the suppression of cell proliferation, nucleic acid synthesis and folate metabolism [2]. However, MTX can induce toxicity in different organs including the kidney, liver, bone marrow and testis [3]. Previous experimental and clinical studies have reported the mechanism by which MTX can induce testicular injury. It includes apoptotic cell death and the release of pro-inflammatory cytokines and reactive oxygen species (ROS) [4]. It was proved that MTX-induced oxidative stress causes damage to the testis morphology, irreversible injury of germ cells, chromosomal alterations and oligozoospermia [5]. Therefore, it is essential to decrease MTX-induced toxicity without interferring with its efficacy.

Herbal medicine with antioxidant potential is considered to be a felicitous strategy to counteract toxicity induced by antineoplastic drugs [6,7,8,9]. *Marrubium alysson* L. (*M. alysson* L.) (Lamiaceae) is common in Egypt along the coast of the Mediterranean Sea [10]. The plant was previously subjected to chemical investigation that resulted in isolation and identification of a number of diterpenes, sterols, flavonoids and phenolic compounds [11]. The potential of flavonoids and phenolics as antioxidants has been studied extensively and a substantial structure-activity relationship of the antioxidant effect has been confirmed [12]. Moreover, *M. alysson* L. was investigated for its biological effect and exhibited potent antioxidant and anti-inflammatory activities [13]. Accordingly, it was selected to be investigated as a promising agent to counteract MTX-induced testicular toxicity.

The target of the current study was to explore the potentiial protective effect of the plant *M. alysson* L. against MTX-induced testicular injury in mice. The efficacy of the crude methanolic extract, polyphenolic fraction, and three selected pure flavonoids (kaempferol, quercetin and rutin) that were detected in the plant was examined. Moreover, the chemical constituents of the methanolic extract of *M. alysson* L. were tentatively determined using liquid chromatography coupled to tandem mass spectrometry (LC-MS/MS). 

## 2. Results

### 2.1. In Vivo Investigation

#### 2.1.1. Effect on Serum Testosterone Level

**Figure 1:**
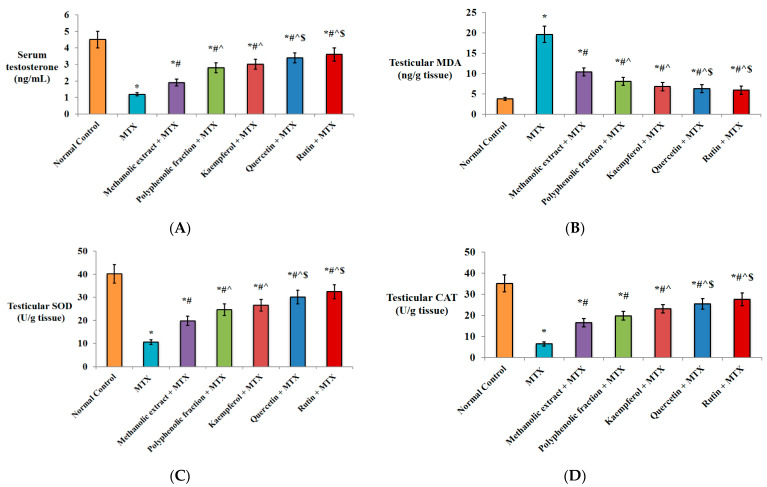
Effect of *M. alysson* L. methanolic extract, its polyphenolic fraction, kaempferol, quercetin and rutin on testicular injury-induced mice. (**A**) Serum testosterone, (**B**) Testicular MDA, (**C**) Testicular SOD level and (**D**) Testicular CAT level. Data are presented as mean ± SD, (n = 8). * Significant versus normal control. # Significant versus MTX. ^ Significant versus methanolic extract. $ Significant versus polyphenolic fraction. *p* value ≤ 0.01.

It is apparent from Figure 1A that single MTX injection significantly decreased the serum testosterone level (↓73.3%) versus the normal control group. Pre-treatment of mice with either methanolic extract or polyphenolic fraction induced a significant increase in the hormonal level (1.5- and 2.3-fold, respectively) compared to the MTX group. Better improvement was observed by pre-treatment with kaempferol (2.5-fold), quercetin (2.8-fold) or rutin (3-fold) versus MTX group. Moreover, the poyphenolic fraction, kaempferol, quercetin and rutin significantly increased the testosterone level (1.5-, 1.6-, 1.8- and 1.9-fold, respectively) versus the *M. alysson* L. methanolic extract group. In comparison with the polyphenolic fraction group, the testosterone level was significantly increased (1.2- and 1.3-fold) by pre-treatment with quercetin and rutin, respectively (Figure 1A).

#### 2.1.2. Antioxidant Activity

Statistical analysis revealed that the testicular malondialdehyde (MDA) content was significantly elevated by single MTX injection (5.1-fold) versus normal control. Oral administration of methanolic extract prior to MTX injection antagonized the MTX-induced elevation of MDA in testicular tissues. The polyphenolic fraction significantly decreased MDA (↓58.7%) versus the MTX group. In addition, kaempferol, quercetin and rutin significantly decreased the testicular MDA level (↓65.3%, ↓67.8%, ↓69.9%, respectively) in comparison to the MTX group. A significant decrease was also observed by pre-treatment with *M. alysson* L. polyphenolic fraction (↓22%), kaempferol (↓34.6), quercetin (↓39.4) and rutin (↓43.3) versus pre-treatment with the methanolic extract. Protection of the mice with quercetin and rutin showed a significant reduction in the MDA testicular level (↓22.2% and ↓27.2%, respectively) compared to the polyphenolic fraction (Figure 1B).

Strong evidence of *M. alysson* L. antioxidant activity was obtained from superoxide dismutase (SOD) and catalase (CAT) assays. MTX significantly decreased the testicular SOD and CAT levels (↓73.5, ↓81.4%, respectively) in comparison to the normal control group. However, prior treatment with the methanolic extract of *M. alysson* L. or its polyphenolic fraction resulted in significant elevation in the SOD level by 1.8- and 2.3-fold, respectively, and in the CAT level by 2.5- and 3-fold, respectively, versus the MTX group (Figure 1C,D).

Further elevation in the SOD level was found in mice pre-treated with kaempferol (2.5-fold), quercetin (2.8-fold) and rutin (3-fold) versus the MTX group (Figure 1C). Similar improvement in the CAT levels was achieved by kaempferol (3.5-fold), quercetin (3.9-fold) and rutin (4.2-fold) versus the MTX group (Figure 1D).

In comparison with the metnanolic extract, SOD level was significantly increased (1.3-, 1.5- and 1.6-fold) by pre-treatment with kaempferol, quercetin and rutin, respectively. In the same manner, CAT level was increased (1.4-, 1.5- and 1.7-fold) by pre-treatment with kaempferol, quercetin and rutin, respectively. Quercetin and rutin induced significant elevation in SOD level by 1.2- and 1.3-fold, respectively, and in CAT level by 1.3- and 1.4-fold, respectively, versus the polyphenolic fraction group (Figure 1C,D).

#### 2.1.3. Anti-Inflammatory Activity

**Figure 2:**
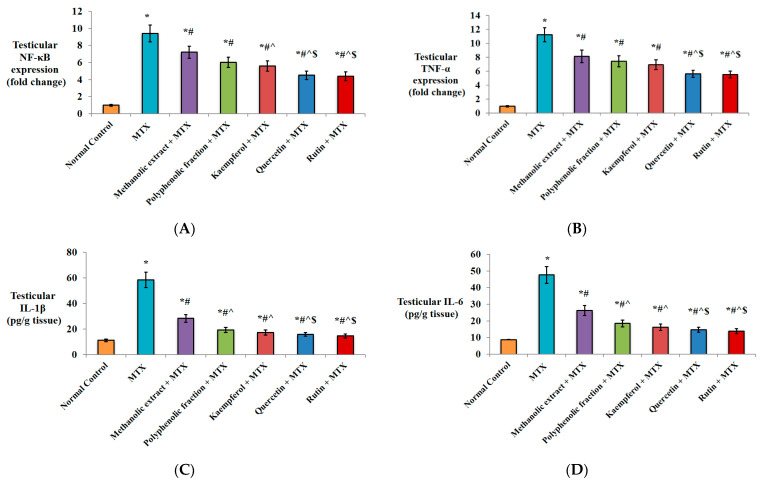
Effect of *M. alysson* L. methanolic extract, its polyphenolic fraction, kaempferol, quercetin and rutin on testicular injury-induced mice. (**A**) Testicular NF-κB mRNA expression, (**B**) Testicular TNF-α mRNA expression, (**C**) Testicular IL-1β level and (**D**) Testicular IL-6 level. Data are presented as mean ± SD, (n = 8). * Significant versus normal control. # Significant versus MTX. ^ Significant versus methanolic extract. $ Significant versus polyphenolic fraction. *p* value ≤ 0.01.

An anflammatory response mediated by single MTX injection was obvious from the significant nuclear factor kappa B (NF-κB) and tumor necrosis factor-alpha (TNF-α) up-regulation as well as interleukin-6 (IL-6) and interleukin-1β (IL-1β) elevation in testicular tissues compared to normal control (Figure 2A,B).

Pre-treatment with *M. alysson* L. methanolic extract or its polyphenolic fraction significantly down-regulated NF-κB (↓23.4% and ↓36.1%, respectively) compared to the MTX group. Further down-regulation was achieved by pre-treatment with kaempferol (↓40.4%), quercetin (↓52.1%) and rutin (↓53.1%) relative to only MTX injection. Kaempferol, quercetin and rutin exhibited further down-regulation of NF-κB expression by ↓22.2%, ↓37.5%, and ↓38.8%, respectively, relative to the methanolic extract group. In comparison with polyphenolic fraction pre-treatment, the percentage of NF-κB down-regulation achieved by quercetin and rutin was ↓25.1% and ↓26.6, respectively (Figure 2A).

In comparison with the MTX group, TNF-α was significantly down-regulated (↓27.6% and ↓33.9%) by pre-treatment with *M. alysson* L. methanolic extract and its polyphenolic reaction, respectively. Pre-treated mice with kaempferol, quercetin and rutin revealed greater down-regulation (↓38.3%, ↓50%, and ↓50.8%) relative to MTX alone. Protection with quercetin and rutin significantly down-regulated TNF-α (↓30.8% and ↓32.1%, respectively) versus the methanolic extract and (↓24.3% and ↓25.7%, respectively) versus the polyphenolic fraction (Figure 2B).

A remarkable increase in the level of IL-1β and IL-6 (5.2- and 5.4-fold, respectively) was recorded in MTX group versus normal control mice (Figure 2C,D). Conversely, pre-treated mice with *M. alysson* L. methanolic extract displayed a significant decline in IL-1β and IL-6 testicular concentrations by ↓51.3% and ↓44.9%, respectively, in comparison to the MTX group. The polyphenolic fraction, kaempferol, and quercetin revealed a more pronounced decline in IL-1β (↓66.7%, ↓70.3%, and ↓72.7%, respectively) in comparison to the MTX group. In addition, the levels of IL-6 were significantly decreased to a greater extent by prior treatment with the polyphenolic fraction (↓61.1%), kaempferol (↓65.9%), and quercetin (↓69.1%) versus the MTX group. The most pronounced declines in IL-1β (↓74.9%) and IL-6 (↓70.8%) testicular levels were noticed in the rutin-receiving group when compared to the MTX group (Figure 2C,D).

In comparison with protection by *M. alysson* L. metnanolic extract, the IL-1β levels were significantly decreased by ↓31.7%, ↓39.1%, ↓44.0%, and ↓48.6% in the polyphenolic fraction, kaempferol, quercetin and rutin groups, respectively. Similarly, the IL-6 levels were decreased (↓29.4%, ↓38.2%, ↓43.9% and ↓46.9) by pre-treatment with the polyphenolic fraction, kaempferol, quercetin and rutin, respectively. Quercetin and rutin induced a significant decline in the IL-1β levels by ↓18.0% and ↓24.7%, respectively, and in the IL-6 levels by ↓20.5% and ↓24.8%, respectively, versus the polyphenolic fraction (Figure 2C,D).

#### 2.1.4. Effect on Apoptotic Markers

**Figure 3:**
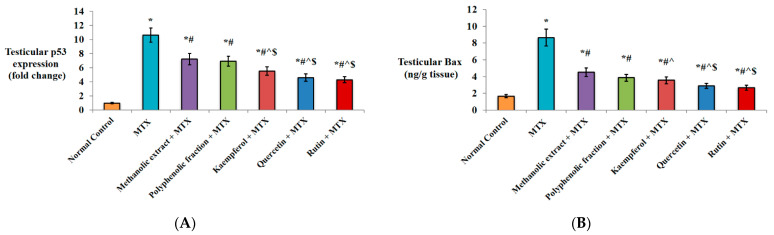

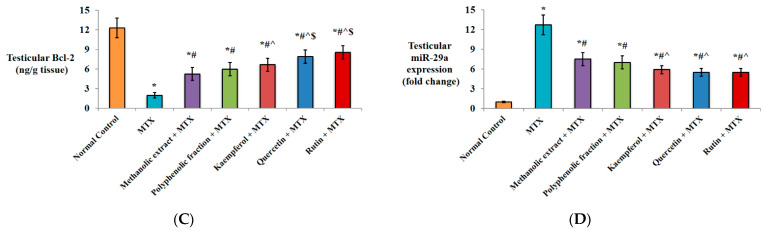
Effect of *M. alysson* L. methanolic extract, its polyphenolic fraction, kaempferol, quercetin and rutin on testicular injury-induced mice. (**A**) Testicular p53 mRNA expression, (**B**) Testicular Bax level, (**C**) Testicular Bcl-2 level and (**D**) Testicular miRNA-29a expression. Data are presented as mean ± SD, (n = 8). * Significant versus normal control. # Significant versus MTX. ^ Significant versus methanolic extract. $ Significant versus polyphenolic fraction. *p* value ≤ 0.01.

As illustrated in Figure 3A, MTX injection resulted in the significant up-regulation of p53 testicular expression by 10.6-fold compared to normal healthy mice. Remarkable decrease in p53 gene expression by ↓32.1% and ↓34.9% was noticed in *M. alysson* L. methanolic extract and polyphenolic fraction groups, respectively, in comparison to the MTX group. More prominent down-regulation was found in kaempferol (↓48.1%), quercetin (↓56.6%) and rutin (↓59.4%) pre-treated mice versus the MTX group. Protection of the mice with kaempferol, quercetin and rutin significantly down-regulated p53 (↓23.6%, ↓36.1% and ↓40.3% respectively) versus the methanolic extract and (↓20.3%, ↓33.3% and ↓37.7%, respectively) versus the polyphenolic fraction (Figure 3A).

Protection of the mice with oral administration of *M. alysson* L. methanolic extract or its polyphenolic fraction showed significant reduction in Bax testicular level (↓47.7% and ↓55.4%, respectively) compared to unprotected mice. Kaempferol, quercetin and rutin showed a more evident decline (↓58.8%, ↓66.6% and ↓69.1%, respectively) compared to only MTX-injected mice. Oral administration of quercetin and rutin significantly decreased Bax testicular level (↓36.1% and ↓40.9%, respectively) versus the methanolic extract and (↓25.1% and ↓30.8%, respectively) versus the polyphenolic fraction (Figure 3B). 

Figure 3C shows that the induction of testicular injury by MTX significantly decreased the Bcl-2 level by↓83.9% in comparison to the normal control group. Furthermore, protection against MTX by *M. alysson* L. methanolic extract or its polyphenolic fraction significantly increased the Bcl-2 testicular level (2.6- and 3.1-fold) versus unprotected mice. More outstanding elevation was observed by protection with kaempferol (3.3-fold), quercetin (4-fold) and rutin (5.5-fold) compared to unprotected mice. In comparison with protection by the metnanolic extract, Bcl-2 level was significantly increased (1.3-, 1.5- and 1.6-fold) by pre-treatment with kaempferol, quercetin and rutin, respectively. Quercetin and rutin induced significant elevation in Bcl-2 level by 1.3- and 1.4-fold, respectively, versus the polyphenolic fraction group (Figure 3C).

#### 2.1.5. Effect on miRNA-29a Expression 

A statistically significant increase in miRNA-29a testicular expression (12.7-fold) was recorded in MTX group versus normal control mice. Pre-treatment with *M. alysson* L. methanolic extract or its polyphenolic fraction significantly down-regulated miRNA-29a (↓40.9% and ↓44.8%, respectively) compared to the MTX group. Further down-regulation was achieved by pre-treatment with kaempferol (↓53.5%), quercetin (↓56.6%) and rutin (↓56.6%) relative to only MTX injection. In comparison with the methanolic extract pre-treatment, kaempferol significantly down-regulated miRNA-29a by ↓21.3%, while quercetin and rutin achieved the same percentage of down-regulation (26.6%) (Figure 3D).

#### 2.1.6. Histopathological Findings

**Figure 4:**
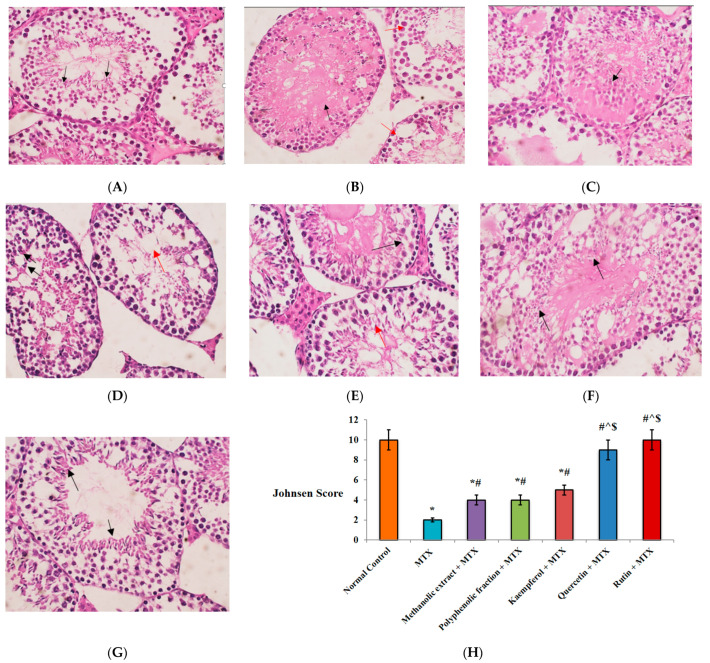
Photomicrographs of testicular sections from healthy and testicular injury-induced mice and effect of *M. alysson* L. methanolic extract, its polyphenolic fraction, isolated kaempferol, quercetin and rutin on Johnsen score in testicular-injury-induced mice. (**A**) Normal control group showing full spermatogenesis in all tubules, up to the level of spermatozoa (black arrows). (**B**) MTX group showing some hyalinized tubules (40%) (black arrow), with other tubules showing significant reduction in spermatogenesis, with few spermatocytes (red arrows). (**C**) Methanolic extract + MTX group showing hyalinized tubules with markedly disturbed spermatogenesis, with detected spermatocytes only. (**D**) Polyphenolic fraction + MTX group showing full spermatogenesis in some tubules (60%), but with few spermatozoa (black arrows), other tubules show marked decreased spermatogenesis (red arrows). (**E**) Kaempferol + MTX group showing absent germinal cells in some tubules (30%), with hyalinized lumen and with only Sertoli cells (black arrow), while other tubules show spermatogenesis, to the level of early spermatids (red arrows). (**F**) Quercetin + MTX group demonstrated complete spermatogenesis in all tubules, reaching the level of spermatids (black arrows), despite the disturbed lining epithelium and the hyalinized tubal lumens. (**G**) Rutin + MTX group showing full spermatogenesis in all tubules, up to level of spermatids (black arrows) (H&E, 40×). (**H**) Johnsen Score. Data are presented as mean ± SD, (n = 8). * Significant versus normal control. # Significant versus MTX. ^ Significant versus methanolic extract. $ Significant versus polyphenolic fraction. *p* value ≤ 0.01.

The normal architecture of seminiferous tubules with full spermatogenesis was seen in testicular sections from normal healthy mice (Figure 4A). However, single MTX injection induced a remarkable decrease in the Johnsen score compared to normal testicular sections (Figure 4H) and showed hyalinized tubules with a significant decrease in spermatogenesis (Figure 4B). These histopathological changes were well alleviated by administration of *M. alysson* L. and selected pure components before MTX injection. A two-fold increase in the Johnsen score versus MTX was achieved by *M. alysson* L. methanolic extract and its polyphenolic fraction (Figure 4H). In addition, the methanolic-extract-receiving mice revealed hyalinized tubules with only detected spermatocytes (Figure 4C), while polyphenolic-fraction-administered mice showed full spermatogenesis in 60% of the tubules, with few spermatozoa and markedly decreased spermatogenesis in other tubules (Figure 4D). 

Sections from mice pre-treated with kaempferol and quercetin a showed significant increase (2.5- and 4.5-fold, respectively) in Johnsen score in comparison to the MTX group (Figure 4H). Moreover, kaempferol revealed only 30% of seminiferous tubules with hyalinized lumen and absent germinal cells, while other tubules showed spermatogenesis, to the level of early spermatids (Figure 4E). Quercetin-induced full spermatogenesis reached the level of spermatids in all tubules but with hyalinized lumens (Figure 4F).

The rutin group displayed the most outstanding improvement in histopathological changes, where the Johnsen score was reversed to its normal value. Full spermatogenesis in all tubules reached the level of spermatids with normal lining epithelium and the absence of hyalinization evident (Figure 4G,H). Quercetin and rutin induced significant elevation in the Johnsen score by 2.25- and 2.5-fold versus the methanolic extract and the polyphenolic fraction groups, respectively (Figure 4H).

### 2.2. LC-ESI-TOF-MS/MS Analysis of Marrubium Alysson L.

**Figure 5:**
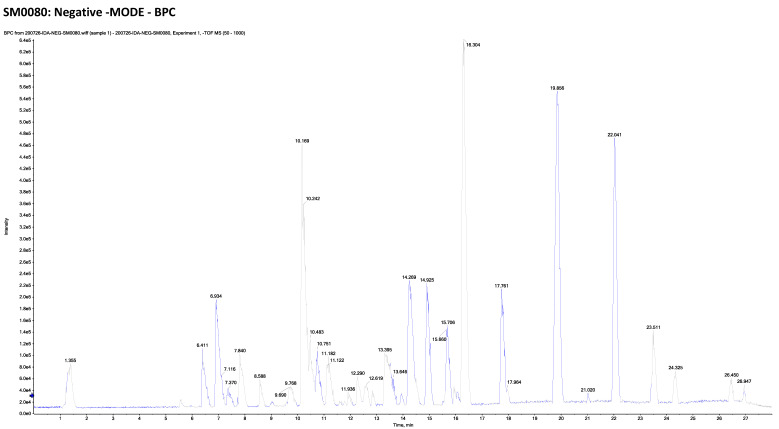
Chromatogram of methanolic extract of *M. alysson* L. in negative ion mode.

**Figure 6:**
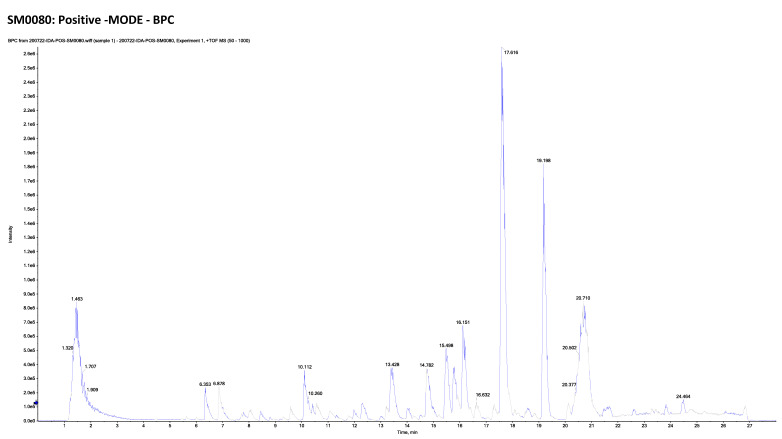
Chromatogram of methanolic extract of *M. alysson* L. in positive ion mode.

The combination of liquid chromatography and tandem mass spectrometry (LC-MS/MS) is an effective method for identifying natural metabolites that could be promising therapeutic agents. In the current study, the LC-MS/MS of *M. alysson* L. methanolic extract is presented (Figure 5 and Figure 6). 

This metabolomics profiling demonstrated 40 compounds, including 23 compounds detected in the positive ion mode and 17 compounds detected in the negative ion mode (Table 1).

The detected compounds were from different classes such as alkaloids, catechins, flavonoids and their glycosides, phenylethanoid glycosides, coumarins and their glycosides, amino acids, and other miscellaneous compounds (Table 1, Figure S1).

### 2.3. Total Phenolic and Total Flavonoid Content Determination

The total phenolic content was found to be 53.21 mg/g dry extract (calculated as gallic acid equivalent), while the total flavonoid content was determined as 27.37 mg/g dry extract (calculated as rutin equivalent).

### 2.4. Quantitative Estimation of Kaempferol, Quercetin and Rutin Using HPLC

HPLC chromatogram revealed that kaempferol is a major component of the extract followed by quercetin and rutin. Accordingly, these three compounds were selected to be determined quantitatively in the methanolic extract of *M. alysson* L. Validation of the method was performed, and the results were found to be satisfactory, as outlined in the following subsections.

#### 2.4.1. Analytical Solution Stability

The described analysis method was repeated under different storage conditions, at 4 °C for ten days and at room temperature for two days. The outcomes were compared to those of freshly prepared standard solutions. No significant difference was noticed, indicating the stability of the analytical solution.

#### 2.4.2. Linearity

The linearity of the performed HPLC method was ascertained by the analysis of five different concentrations of each standard (kaempferol, quercetin and rutin) each in triplicate. Linear regression data for the calibration curves of the three standard compounds showed a good linear relationship over the concentration range of 10–150, 5–100 and 10–200 µg/mL for kaempferol, quercetin and rutin, respectively. The linear regression equations were y = 10,335.26x − 33,633.67 (R^2^ = 0.99) for kaempferol, y = 11,871.11x − 139,172.6 (R^2^ = 0.99) for quercetin and y = 11,062.25x − 24,842.13 (R^2^ = 0.99) for rutin.

#### 2.4.3. Precision of the System

The precision of the system was assured from the low values of relative standard deviation (%RSD) calculated after repeated application (triplicate) of a selected concentration of the standard compounds (100 µg/mL). The %RSD was found to be 0.72, 0.71 and 0.52 for kaempferol, quercetin and rutin, respectively.

#### 2.4.4. Method Precision

To ensure validation of the method, 100 µg/mL of the methanolic extract of *M. alysson* were injected (four times). The %RSD was calculated as 0.91, 1.44 and 0.66 for kaempferol, quercetin and rutin, respectively.

#### 2.4.5. Limits of Detection and Quantification

Limits of detection of the investigated flavonoids were calculated according to the equation 3 σ/S and found to be 0.5, 0.5 and 0.7 µg/mL for kaempferol, quercetin and rutin respectively. Furthermore, the quantification limits were calculated using the equation 10/S and were found as 1.60, 1.63, 2.4 for kaempferol, quercetin and rutin, respectively, where σ is the standard deviation of the response, and S is the slope of the calibration curve.

#### 2.4.6. Sample Analysis

All the tested validation parameters were found to be reasonable. Accordingly, the suggested method was applied to calculate the concentration of the three mentioned flavonoids in the methanolic extract of *M. alysson*. The method was repeated four times and the concentrations were calculated based on the regression equation. Kaempferol, quercetin and rutin were found to be 2.7, 0.29 and 0.71 mg/g of the plant extract, respectively.

## 3. Discussion

Systemic chemotherapeutic agents might affect different body organs and cause life-threatening adverse effects [48]. Testicular toxicity is a serious side effect of MTX chemotherapy; therefore, protection of the reproductive systems and their germinal cells is important during the use of MTX [49,50]. This study aimed to identify the chemical constituents of *M. alysson* L. methanolic extract and to investigate its potential protection against mice’s testicles from MTX-induced damage. The protective effects of *M. alysson* L., polyphenolic fraction and three of its selected pure flavonoids (i.e., kaempferol, quercetin, and rutin) were also addressed. In addition, the possible mechanisms behind the potential protective effects were studied. 

Testicular tissues are at high risk of developing oxidative-stress-induced damage due to their great content of unsaturated fatty acids, high rate of mitochondrial oxygen consumption and rapid cell division. Additionally, pollutants from the environment, diet, and X-ray and chemotherapy exposure can exaggerate this injury [51]. In the present study, single MTX injection caused histopathological injury of the testis along with a significant reduction in the spermatogenesis Johnsen score and serum testosterone level. These pathological hallmarks could be developed by oxidative stress, as evidenced by a rise in lipid peroxidation byproducts and decline in antioxidant enzyme levels in testicular tissues. Previous studies have reported MTX-induced sperm abnormalities and infertility through lipid peroxidation and ROS generation [52,53]. 

Natural antioxidants represent a valuable strategy to protect reproductive tissues from drug-induced adverse effects [54]. Oral administration of *M. alysson* L. extract prior to MTX injection induced elevation in the testicular levels of the antioxidant enzymes SOD and CAT and a reduction in the level of the lipid peroxidation indicator MDA. Better antioxidant activity was exerted by *M. alysson* L. polyphenolic fraction. A previous investigation reported the antioxidant activity of *M. alysson* L. in obese rabbits [13]. Since isolated bioactive compounds from natural products provide numerous opportunities for the development of new therapeutic drugs, scientific investigations usually focus on certain bioactive compounds rather than a whole herb extract [55]. This supports our findings, wherein more potentiated antioxidant capacity was achieved by pre-treatment with the pure compounds kaempferol, quercetin and rutin as described in previous reports [56,57,58].

Activation of immune cells and macrophages stimulates the generation of pro-inflammatory cytokines that can regulate the immune and inflammatory response [59]. Inflammation and oxidative stress are interrelated since oxidative stress is involved in NF-κB activation and the latter stimulates oxidative stress [60]. Here, oxidative stress and inflammation are involved in the pathogenesis of testicular injury induced by MTX injection. A significant boost in the testicular level of IL-1β and IL-6 and significant NF-κB and TNF-α mRNA up-regulation are evidence for testicular injury in MTX-injected group. MTX was reported to activate NF-κB signaling pathway and its nuclear localization through activation of Toll-like receptor 4 (TLR4). NF-κB translocation into the nucleus can enhance production of inflammatory mediators such as TNF-α, IL-1β and IL-6 [61,62].

The anti-inflammatory activity of *M. alysson* L. methanolic extract and its polyphenolic fraction has been proved in this study, manifested by improved histopathological changes in testicular tissues that were accompanied by a decline in the testicular levels of IL-1β and IL-6 and significant NF-κB and TNF-α mRNA down-regulation. This interpretation could be supported by Edziri et al. [63], who determined the anti-inflammatory activity of *M. alysson* L. methanolic extract by the carrageenan-induced paw edema assay in rats and referred to its butyrylcholinesterase inhibitory activity. Furthermore, *M. alysson* L. can exert this beneficial effect by decreasing the secretion of serotonin, histamine, prostaglandins and kinin-like substances [64]. Strong antioxidant and anti-inflammatory properties of polyphenolic components have been extensively reported [65]. These well-documented properties support our revealed anti-inflammatory activity of the *M. alysson* L. polyphenolic fraction.

Kaempferol, quercetin and rutin detected in *M. alysson* L. exhibited significant anti-inflammatory activity to preserve testicular functions in mice injected with MTX. The histopathological findings are strongly linked to this activity, where mice pre-treated with kaempferol and quercetin showed significant elevation in Johnsen score relative to the MTX group. Quercetin induced full spermatogenesis up to the level of spermatids in all tubules but with hyalinized lumen. Rutin displayed the most pronounced improvement in histopathological changes, where the Johnsen score was reversed to its normal value and full spermatogenesis in all tubules was evident. Similar findings have been revealed by Zhang et al. [66], who explained the kaempferol anti-inflammatory mechanism through the inhibition of TLR4/myeloid differentiation factor 88 (MyD88)-mediated NF-κB signaling pathway. Kaempferol and quercetin were reported to increase the mRNA and protein expression of nuclear factor (erythroid-derived 2)-like 2 (Nrf2)-regulated genes in HepG2-C8 cells [67,68]. Previous studies on RAW 264.7 cells and rat paw oedema confirmed the anti-inflammatory activity of rutin through different mechanisms [69,70].

The current results suggest that the mechanisms of MTX-induced apoptosis in testicular tissue are similar to those reported in previous research articles. Apoptosis is considered an important factor to the pathophysiology of testicular damage caused by MTX [53,71]. Therefore, the inhibition of MTX-induced apoptosis is a potential therapeutic target to prevent testicular toxicity. The antiapoptotic protein Bcl-2 can inhibit the apoptotic mitochondrial pathway by suppressing Bax oligomerization and cytochrome c release [72]. It was found in this study that MTX induced apoptotic cell death in testicular tissue through the pathways of p53, Bax, and Bcl-2. The generation of ROS by MTX could be the cause of the developed apoptosis since oxidative stress causes caspase activation through impairment of the mitochondria functions and subsequent cytochrome c release [73].

High flavonoid and phenolic content of several natural products contribute to their protective effects against MTX exposure through the inhibition of various apoptotic pathways and DNA damage [74,75]. The methanolic extract of *M. alysson* L. and its polyphenolic fraction exerted their protective effect through the inhibition of mitochondria-dependent apoptosis, as displayed from the down-regulation of p53 testicular expression accompanied with a decline in Bax and elevation of Bcl-2 testicular levels. In addition, our results documented the ability of the phenolic constituents of *M. alysson* L. to protect the reproductive tissue from MTX-induced apoptosis. These findings are consistent with Wang et al. [76], who reported the inhibition of inflammatory and oxidative-stress-induced apoptosis by kaempferol to protect against cerebral ischemia reperfusion injury. Quercetin has been reported to inhibit apoptosis in human umbilical vein endothelial cells (HUVECs) *via* downregulation of activator protein 1 (AP-1) and NF-κB signaling pathway [77]. High-glucose-induced cardiomyocyte injury has been alleviated by rutin treatment through inhibition of apoptosis and endoplasmic reticulum stress [78].

There are several scientific investigations that have explained the role of miRNA-29a in inducing cell apoptosis in various models including dextran-sodium-sulfate-induced ulcerative colitis [79], myocardial ischemia/reperfusion [80] and hyperoxia-induced bronchopulmonary dysplasia [81]. Up-regulation of the miRNA-29 family expression including miRNA-29a was parallel with apoptosis in germ cells upon administration of estradiol benzoate and doxorubicin [82,83]. Activation of inflammatory signaling pathways and the development of oxidative stress can increase miRNA-29a expression and induce apoptosis [84]. These findings support the results of this work, where miRNA-29a up-regulation was parallel to the up-regulation of p53 testicular expression accompanied with the elevation of Bax and decline in Bcl-2 testicular levels in the MTX group. 

Several natural compounds can regulate the expression profile of numerous miRNAs that are involved in different pathological disorders [85,86]. The methanolic extract of *M. alysson* L. and the phenolic compounds presented, for the first time, a significant down-regulation in testicular miRNA29-a expression, indicating their ability to reduce the testicular toxicity induced by MTX. This down-regulation coincided with their proved antioxidant, anti-inflammatory and anti-apoptotic activities. The harmony of our results is strongly related to previous publications which suggested that the suppression of miRNA-29a expression improved different disorders [79,80,81].

*M. alysson* L. was inspected using LC-ESI-TOF-MS/MS, the chromatograms of methanolic extract in positive and negative mode ion are exhibited in Figure 5 and Figure 6. The results are illustrated in Table 1 and Figure S1. Tentative characterization of the extract components was acquired through comparison of the chromatographic behavior, m/z values and fragmentation pattern with those previously mentioned in the literature. Accordingly, 40 hits were recognized, where the majority of the identified compounds were flavonoids (16 compound). Previous reports on the chemical constituents of *M. alysson* L. revealed the isolation of a number of flavonoids including chrysoeriol, acacetin, acacetin-7-glucoside, diosmetin, diosmetin-7-rhamnoside, luteolin, luteolin-7-rutinoside, quercetin in addition to apigenin and its 7- glycosides [11,87].

## 4. Materials and Procedures

### 4.1. Plant Material

*M. alysson* L. was gathered in Alexandria (Burg El-Arab) during April 2020. The collected plant was identified at the Faculty of Science, Suez Canal University. A voucher specimen (M.A. 2020) was placed at the Department of Pharmacognosy herbarium (Faculty of Pharmacy, Suez Canal University, Ismailia, Egypt).

### 4.2. Preparation of Plant Crude Extract

*M. alysson* L. whole plant (one Kg) was dried in shade for two weeks. The *M. alysson* L. plant was then finely pulverized and extracted by MeOH (3 × L) at ordinary temperature. The filtrates were compiled together and distilled under vacuum with a rotary evaporator (BÜCHI Labortechnik AG, Flawil, Switzerland). The yield of the extract was 193 g; accordingly, each gram of the powdered plant gives 0.193 g of dry extract.

### 4.3. Preparation of Phenolic Extract

The phenolic portion was prepared by treatment of 500 g of the pulverized *M. alysson* L. with 5% aqueous solution of Na_2_CO_3_, left for 1 h, followed by filtration, and then rinsed with distilled water. Distilled water was used for the dilution of the filtrate, which was then neutralized by HCl. After that, it was partitioned consecutively between CHCl_3_, EtOAc and *n*-BuOH. All extracts were compiled together followed by concentration under a vacuum. 

### 4.4. Chemicals

Kaempferol, quercetin and rutin were purchased from Sigma-Aldrich (Egypt); all the solvents used for the extraction process were obtained from Merck (Egypt).

### 4.5. Determination of Total Phenolic and Total Flavonoid Contents:

Determination of the total phenolics of *M. alysson* L. extract was performed spectrophotometrically as previously described [88]. Milton Roy, Spectronic 1201, Houston, TX, USA was used for the measurement. The result was calculated as gallic acid equivalents (mg/g dry extract). Total flavonoids were estimated according to the reported method [89]. Measurement was performed at λ 420 nm. The result was calculated as rutin equivalents (mg/g dry extract).

### 4.6. HPLC-DAD Quantitative Analysis 

#### 4.6.1. Instrumentation

The analysis was carried out using a Waters 2690 Alliance HPLC system outfitted with a Waters 996 photodiode array detector and a column C18 Inertsil ODS (with dimensions equal 4.6 × 250 mm and a particle size equal 5 µM).

#### 4.6.2. Operating Conditions

The mode of elution was gradient using mobile phase 0.1% phosphoric acid in water: acetonitrile. The flow rate was 1 mL/min at ambient temperature and detection was performed at λ 280 nm.

#### 4.6.3. Standard and Sample Preparation

Stock solutions of the different standards were prepared by dissolving in methanol, each were filtered using 0.22 µM syringe filter, serial dilutions were obtained, and 10 µL of each of the prepared standard solutions were injected. The methanolic extract of the plant was prepared by accurately weighting 1 g, dissolved in methanol and sonicated for 15 min, filtered using a 0.22 µM Nylon syringe filter, and the volume was adjusted and then 10 µL was used for injection. 

### 4.7. Metabolomic Profiling by LC/MS/MS

The metabolomics profiling was performed according to a previously reported method [19,88]. Briefly, the used mobile phase consisted of DI water:methanol:acetonitrile (2:1:1). A measure of 1 mL of this mobile phase was blended with 50 mg dry methanolic extract of *M. alysson* vortex applied (2 min). Ultra-sonication for 10 min and centrifugation for another 10 min at 10,000 rpm were performed. A measure of 50 µL of the stock was diluted using 1000 µL reconstitution solvent. Accordingly, the concentration used for injection was 1 µg/µL. The injected volume was 25 µLs (in both positive and negative modes). Blank sample consisted of 25 µL of mobile phase working solution. The used mobile phases were: (A) a 5 mM ammonium formate buffer pH 3 plus 1% methanol for positive TOF MS mode; (B) a 5 mM ammonium formate buffer pH 3 plus 1% methanol for negative TOF MS mode; and (C) a 5 mM ammonium formate for both modes. The pre-columns were in-line filter discs (Phenomenex, 0.5 µM × 3.0 mm) and the column was X select HSS T3 (Waters, 2.5 m, 2.1 × 150 mm) and flow rate was 0.3 mL/min. Data processing was obtained through MS-DIAL3.52. The subsidiary standards were applied for extraction of the peaks from total ion chromatogram using MasterView. The signal-to-noise ratio of features must be more than 5, and the sample-to-blank ratio feature intensities must be larger than 3. Characterization of the compounds was possible via accurate mass measurements, MS/MS data, seeking specific spectral libraries and public depots for MS-based metabolomic analysis (MassBank NORMAN, MassBank MoNA, PubChem), retention times along with comparing the data with those published in the literature. Additionally, the mass error was calculated based on the following equation: [(Observed mass − calculated mass) ÷ calculated mass] × 1,000,000 [90].

### 4.8. In Vivo Biological Study 

#### 4.8.1. Animals

Fifty-six male Swiss albino mice, 6–8 weeks old and weighing 15–20 g, were purchased from the Egyptian Organization for Biological Products and Vaccines (Vacsera, Giza, Egypt). 

For acclimatization, mice were kept in clean cages for one week and were provided with free access to a standard diet and water. The instructions for using and caring for experimental animals were followed. The experimental procedures were approved by the Research Ethical Committee, Faculty of Pharmacy, Suez Canal University, Egypt (201910M1).

#### 4.8.2. Study Design 

Eight healthy mice were given a mixture of distilled water/DMSO (2:1) by oral gavage as a vehicle daily for ten days and considered the normal control group. The remaining mice were divided into six groups (each = 8) as follows: MTX group: mice were injected i.p. with a single dose of 20 mg/kg MTX at the fifth day [91]. Methanolic extract + MTX group: mice received 100 mg/kg methanolic extract of *M. alysson* L. every day for ten days by oral gavage with single MTX dose at the fifth day [92]. Polyphenolic fraction + MTX group: mice were given 100 mg/kg *M. alysson* L. polyphenolic fraction every day for ten days by oral gavage with single MTX dose at the fifth day [93]. Kaempferol + MTX group: mice received 10 mg/kg isolated kaempferol every day for ten days by oral gavage with single MTX dose at the fifth day [94]. Quercetin + MTX group mice were given 10 mg/kg isolated quercetin every day for ten days by oral gavage with single MTX dose at the fifth day [95]. Rutin + MTX group: mice received 10 mg/kg isolated rutin every day for ten days by oral gavage with single MTX dose at the fifth day [96]. The methanolic extract, polyphenolic fraction and the three selected compounds were dissolved in distilled water/DMSO mixture (2:1).

At the end of treatment, mice were euthanized under ketamine anesthesia and blood was collected *via* cardiac puncture in plain vacutainers for serum separation. The testes were immediately excised, decapsulated, and divided longitudinally into two sections. The first section was kept frozen at −80 °C for biochemical analysis and the second section was fixed into formalin solution for histopathological examination.

#### 4.8.3. Biochemical Assays

##### Serum Level of Testosterone

The testosterone level (Cat. No. MBS263193) was determined in serum by ELISA kit (MyBioSource, San Diego, CA, USA).

##### Evaluation of Testicular Oxidative Stress Level

MDA (Cat. No. MBS741034), SOD (Cat. No. MBS034842) and CAT (Cat. No. MBS160589) were determined in testicular tissue homogenate samples using ELISA kits obtained from MyBioSource Co. (San Diego, CA, USA).

##### Evaluation of Inflammation and Apoptosis Biomarkers

IL-1β (Cat. No. MBS701092), IL-6 (Cat. No. MBS762321), Bax (Cat. No. MBS763832) and Bcl-2 (Cat. No. MBS2512543) were determined in homogenate samples of testicular tissues using ELISA kits obtained from MyBioSource Co. (San Diego, CA, USA).

##### Quantitative Real-Time Polymerase Chain Reaction for Expression of NF-κB, TNF-α, p53, and miRNA-29a

The quantitative determination of the gene expression levels of NF-κB, TNF-α, p53, and miRNA-29a was performed according to the method described by Eltamany et al. [97]. Total RNA, including miRNA, was extracted from testicular tissues by Qiagen miRNeasy Mini kit (Cat. No. 217004) (Qiagen, Hilden, Germany). The purity and concentration of extracted RNA were determined by NanoDrop spectrophotometer (Thermo Fisher Scientific, Waltham, MA, USA). The expression levels of NF-κB, TNF-α, p53 and miRNA-29a were determined in the testicular tissue using GoTaq^®^ 1-Step RT-qPCR System (Promega, Madison, WI, USA). β-actin was used as an internal control for NF-κB, TNF-α, and p53, while U6B small nuclear RNA (RNU6B) was the endogenous control for miRNA-29a. The annealing temperatures and primers are shown in Table S1. 

One-step RT-qPCR reaction involved reverse transcription at 37 °C for 15 min and inhibition of reverse transcriptase enzyme at 95 °C for 10 min followed by 40 cycles for amplification. The 20 µL reaction was composed of 10 µL GoTaq^®^ qPCR master mix, 4 µL extracted RNA, 1 µL of each primer, 0.4 µL GoScript™ RT mix, 0.31 µL supplemental CXR reference dye, and 3.29 µL nuclease-free water. Each cycle of amplification involved denaturation at 95 °C for 10 s, annealing for 30 s, and extension at 72 °C for 30 s. The reactions were carried out with a StepOnePlus™Real-Time PCR thermal cycling instrument (Applied Biosystems, Waltham, MA, USA). ΔΔCt was calculated and the results were expressed as the mean fold change relative to the normal control group.

#### 4.8.4. Histopathological Examination

Paraffin blocks of testis tissues were formed. Five micrometers of testis sections were stained with hematoxylin and eosin (H&E). Images were recorded by an Olympus microscope (Shinjuku, Japan) equipped with a spot digital camera, and MATLAB software. For Johnsen’s scoring system, seminiferous tubules are classified into ten scores according to their histological features. Score 1: absence of seminiferous epithelium; Score 2: absence of germinal cells, only Sertoli cells are present; Score 3: only spermatogonia are present; Score 4: few spermatocytes are present; Score 5: many spermatocytes are present; Score 6: few early spermatids are present; Score 7: many early spermatids are present; Score 8: less than five spermatozoa per tubule, few late spermatids; Score 9: slightly impaired spermatogenesis, many late spermatids, disorganized epithelium; and Score 10: full spermatogenesis [92].

#### 4.8.5. Statistical Analysis

The results were analyzed by SPSS version 21.0 (IBM, Armonk, NY, USA), and values were presented as mean ± SD. Differences between groups were observed by using a one-way analysis of variance (ANOVA) test followed by Bonferroni’s post hoc multiple comparison test. *p* value ≤ 0.01 was considered significant.

## 5. Conclusions

MTX-induced testicular injury was alleviated by pre-treatment with *M. alysson* L. methanolic extract. Moreover, further protection was achieved by its polyphenolic fraction. This was mediated through the inhibition of apoptosis, inflammation and oxidative stress and down-regulation of miRNA-29a testicular expression. The extract’s beneficial effect is credited to its entire phenolic component set. In addition, we referred to the beneficial role of the pure flavonoids kaempferol, quercetin and rutin as significant constituents of *M. alysson* L. in the alleviation of testicular injury induced by MTX in mice. Histopathological improvement of the testicular tissue was noticed by administration of *M. alysson* L. methanolic extract, polyphenolic fraction and selected pure flavonoids (kaempferol, quercetin and rutin) before MTX injection. More research is needed to screen for and investigate the potential protective effects of other *M. alysson* L. active components and to determine the most safe and effective constituents.

## Figures and Tables

**Table 1:** LC-MS/MS metabolic analysis of *M. alysson* L. methanolic extract.

No.	Polarity Mode	Retention Time (min)	Precursor Type	Measured *m*/*z*	Expected or Calculated *m*/*z*	Name	Molecular Formula	Fragments	Ref.
**Alkaloids**									
1	Positive	1.33	[M + H]^+^	138.0540	138.0550	Trigonelline	C_7_H_7_NO_2_	94, 92	[14]
**Catechins**									
2	Positive	5.51	[M + H]^+^	291.0857	291.0869	Catechin	C_15_H_14_O_6_	123, 139, 147	[15]
3	Positive	4.67	[M + H]^+^	291.0880	291.0869	(-)-Epicatechin	C_15_H_14_O_6_	139, 123	[15,16]
Flavines									
4	Positive	5.45	[M + H]^+^	377.1434	377.1461	(-)-Riboflavin	C_17_H_20_N_4_O_6_	377, 243	[17]
**Flavonoids and their glycosides**									
5	Positive	6.62	[M + H]^+^	611.1652	611.1612	Rutin	C_27_H_30_O_16_	609, 300	[18]
6	Positive	14.02	[M + H]^+^	303.0503	303.0505	Quercetin	C_15_H_10_O_7_	301, 151	[19,20]
7	Positive	7.83	[M + H]^+^	273.0744	273.0763	Naringenin	C_15_H_12_O_5_	273,153, 147	[21]
8	Negative	9.16	[M − H]^−^	315.0542	315.0505	Isorhamnetin	C_16_H_12_O_7_	301, 272	[22]
9	Positive	9.84	[M + H]^+^	287.0566	287.0556	Kaempferol	C_15_H_10_O_6_	287, 153	[23]
10	Negative	10.26	[M − H]^−^	299.0583	299.0556	3 5 7-trihydroxy-4′-methoxyflavone (Diosmetin)	C_16_H_12_O_6_	299, 284	[24]
11	Positive	11.02	[M + H]^+^	271.0618	271.0606	Apigenin	C_15_H_10_O_5_	268, 269 179 225, 201, 151	[25]
12	Positive	6.20	[M + H]^+^	449.1082	449.1084	Luteolin-6-C-glucoside (Isoorientin)	C21H20O11	449	[26]
13	Negative	6.67	[M − H]^−^	623.1609	623.1612	Isorhamnetin-3-*O*-rutinoside	C_28_H_32_O_16_	315, 300	[22]
14	Positive	6.76	[M + H]^+^	433.1110	433.1135	Apigenin 8-C-glucoside (Vitexin)	C_21_H_20_O_10_	431, 433, 311	[25,27]
15	Negative	6.86	[M − H]^−^	447.0906	447.0928	Luteolin-7-*O*-glucoside	C_21_H_20_O_11_	447, 285	[28]
16	Negative	6.95	[M − H]^−^	477.1028	477.1033	Isorhamnetin-3-*O*-glucoside	C_22_H_22_O_12_	477	[22]
17	Positive	7.69	[M + H]^+^	447.0930	447.0927	Baicalein-7-*O*-glucuronide	C_21_H_18_O_11_	245	[29]
18	Positive	7.79	[M + H]^+^	433.1108	433.1135	Apigenin-7-*O*-glucoside (Cosmosiin)	C_21_H_20_O_10_	433,271	[30]
19	Negative	7.96	[M − H]^−^	433.1144	433.1135	Naringenin-7-*O*-glucoside (Prunin)	C_21_H_22_O_10_	433, 271	[31]
20	Positive	10.09	[M + H]^+^	579.1768	579.1714	Apigenin 7-*O*-neohesperidoside (Rhoifolin)	C_27_H_30_O_14_	577, 579, 269, 225.	[25]
	**Phenylethanoid glycosides**								
21	Positive	7.71	[M + H]^+^	639.2277	639.2289	Leucosceptoside A	C_30_H_38_O_15_	177	[32]
22	Positive	8.80	[M + H]^+^	653.2425	653.2446	Martynoside	C_31_H_40_O_15_	485, 339, 177	[32]
23	Negative	7.10	[M − H]^−^	769.2575	769.2555	Alyssonoside	C_35_H_46_O_19_	769, 575	[33]
	**Coumarins and their glycosides**								
24	Positive	5.38	[M + H]^+^	179.0334	179.0344	6,7-dihydroxycoumarin-Aesculetin	C_9_H_6_O_4_	77, 133	[34]
25	Positive	7.01	[M + H]^+^	177.0547	177.0552	7-hydroxy-4-methylcoumarin Hymecromone 4-Methylumbelliferon	C_10_H_8_O_3_	177, 77	[35]
26	Negative	2.32	[M − H]^−^	339.0697	339.0716	Esculin	C_15_H_16_O_9_	2. 399, 177	[36]
	**Amino acids**								
27	Negative	1.22	[M − H]^−^	128.0341	128.0348	L-5-Oxoproline (pyroglutamate)amino a	C_5_H_7_NO_3_	130,84	[37]
28	Positive	1.43	[M + H]^+^	130.0867	130.0868	Pipecolate	C_6_H_11_NO_2_	130, 84	[38]
29	Positive	26.93	[M + H]^+^	118.0867	118.0868	Glycine-Betaine-Trimethylglycine	C_5_H_11_NO_2_	118, 58	[39]
	**Miscellaneous**								
30	Negative	1.38	[M − H]^−^	353.0866	353.0873	Chlorogenic Acid	C_16_H_18_O_9_	191, 179	[40]
31	Positive	4.36	[M + H]^+^	190.0495	190.0504	Kynurenic acid	C_10_H_7_NO_3_	190, 144	[41]
32	Negative	1.18	[M − H]^−^	117.0187	117.0188	Succinic acid	C_4_H_6_O_4_	117,73,99	[42]
33	Positive	17.58	[M + H]^+^	123.0450	123.0446	Benzoic acid	C_7_H_6_O_2_	77	[43]
34	Negative	1.87	[M − H]^−^	163.0386	163.0395	3-(4-hydroxyphenyl) prop-2-enoic acid	C_9_H_8_O_3_	165,119	[36]
35	Negative	1.21	[M − H]^−^	133.0126	133.0137	D-(+)-Malic acid	C_4_H_6_O_5_	115,71	[42]
36	Negative	1.17	[M − H]^−^	195.0491	195.0505	Gluconate	C_6_H_12_O_7_	195	[44]
37	Negative	1.36	[M − H]^−^	179.0556	179.0556	D-(-)-Tagatose-Monosaccharide	C_6_H_12_O_6_	179, 89	[45]
38	Negative	4.91	[M − H]^−^	166.0488	166.0504	Pyridoxal	C_8_H_9_NO_3_	94,168	[46]
39	Negative	19.84	[M − H]^−^	277.2186	277.2168	gamma-Linolenic acid	C_18_H_30_O_2_	275, 259, 233	[47]
40	Positive	4.68	[M + H]^+^	183.0655	183.0657	Syringaldehyde	C_9_H_10_O_4_	123, 77	[16]

## Data Availability

Data are available within the article and Supplementary Materials.

## Supplementary Materials

The following are available online at https://www.mdpi.com/article/10.3390/plants11172309/s1, Figure S1: Chemical structures of the identified compounds in methanolic extract of *M. alysson* L. Table S1: Primer sequences and annealing temperatures for the measured genes by real time PCR.
