# Supplementary material for: Marrubium alysson L. Ameliorated Methotrexate-Induced Testicular Damage in Mice through Regulation of Apoptosis and miRNA-29a Expression: LC-MS/MS Metabolic Profiling
Source: Plants (Basel). 2022 Sep 3;11(17):2309. doi: 10.3390/plants11172309 (PMC9460399; doi:10.3390/plants11172309)
Supplement: Supplementary file 1 [file plants-11-02309-s001.zip › Supplementary data Plants-1839763.pdf]

Marrubium alysson L. Ameliorated Methotrexate-Induced Testicular Damage in Mice through Regulation of Apoptosis and miRNA-29a Expression: LC-MS/MS Metabolic Profiling

Additional Experimental Detail

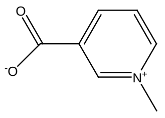

Trigonelline

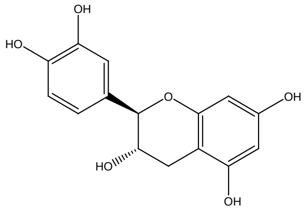

Catechin

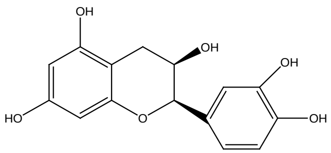

(-)-Epicatechin

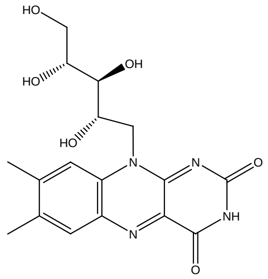

(-)-Riboflavin

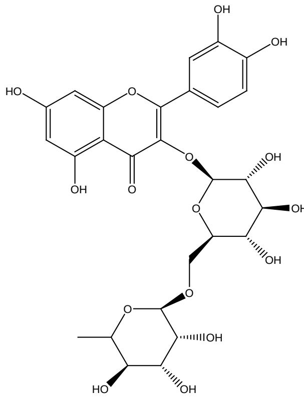

Rutin

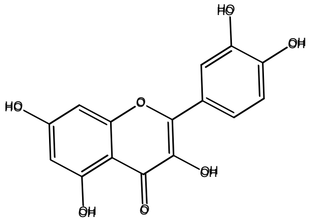

Quercetin

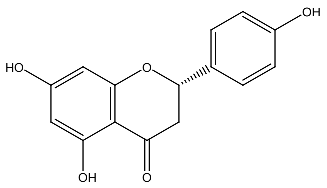

Naringenin

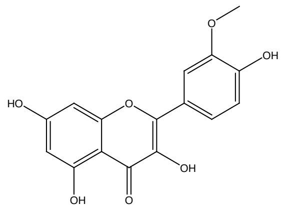

Isorhamnetin

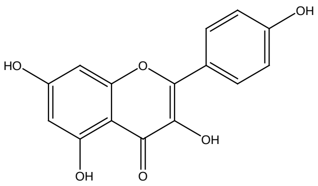

Kaempferol

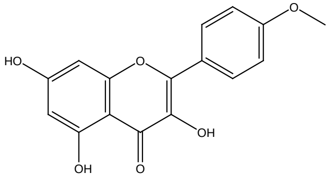

Diosmetin

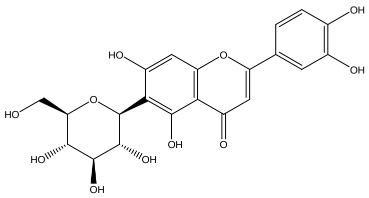

Isoorientin

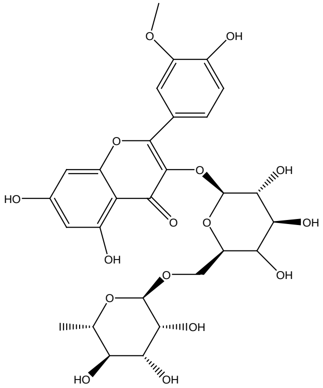

Isorhamnetin-3-O-rutinoside

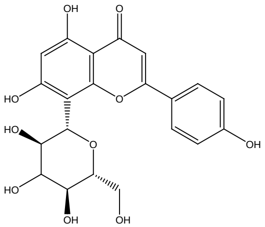

Apigenin 8-C-glucoside

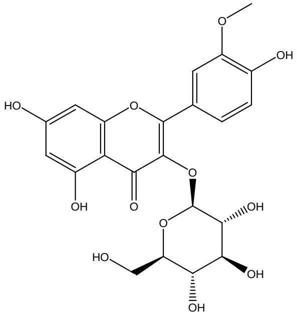

Isorhamnetin-3-O-glucoside

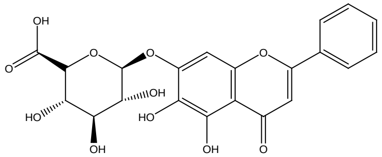

Baicalein-7-O-glucuronide

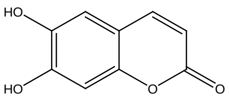

6,7-dihydroxycoumarin

(Aesculetin)

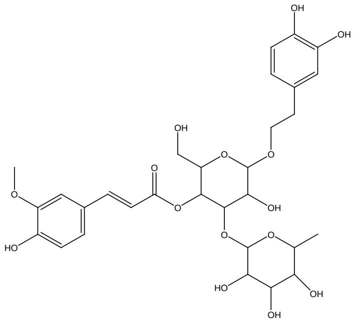

Leucosceptoside A

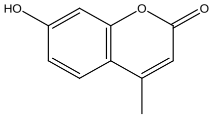

7-hydroxy-4-methylcoumarin

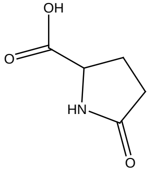

L-5-Oxoproline

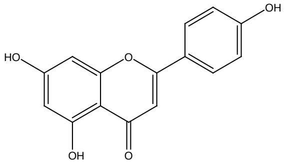

Apigenin

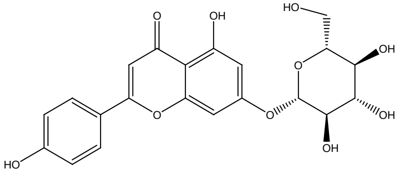

Apigenin-7-O-glucoside

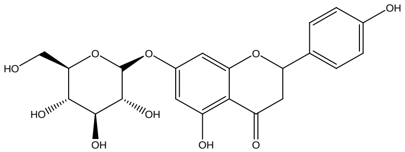

Naringenin-7-O-glucoside

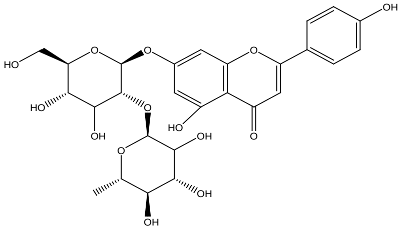

Apigenin 7-O-neohesperidoside

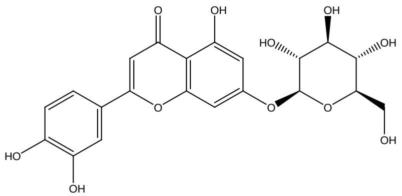

Luteolin-7-O-glucoside

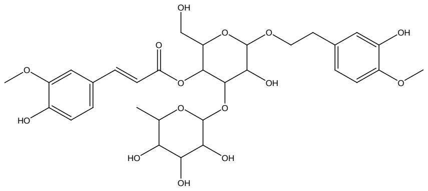

Martynoside

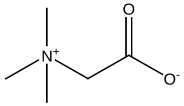

Glycine-Betaine

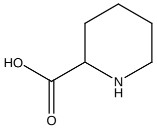

Pipecolate

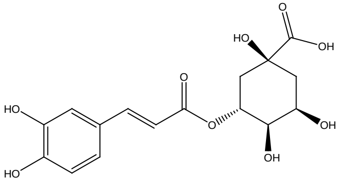

Chlorogenic acid

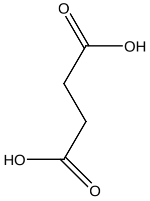

Succinic acid

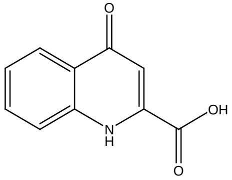

Kynurenic acid

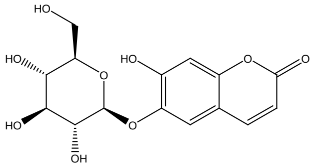

Esculin

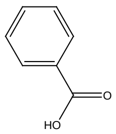

Benzoic acid

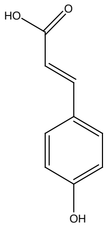

3-(4-hydroxyphenyl)prop-2-enoic acid

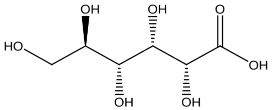

Gluconate

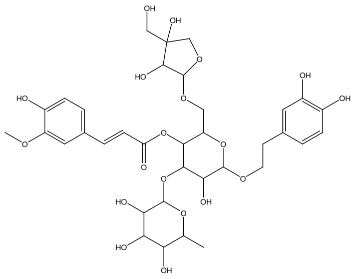

Alyssonoside

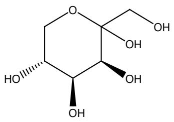

D-(-)-Tagatose

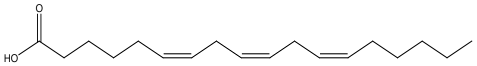

gamma-Linolenic acid

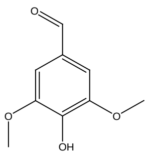

Syringaldehyde

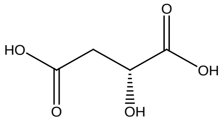

D-(+)-Malic acid

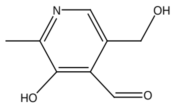

Pyridoxal

Figure S1. Chemical structures of the identified compounds in methanolic extract of *M. alysson* L.

Table S1. Primer sequences and annealing temperatures for the measured genes by real time PCR.

	**Forward Primer**	**Reverse Primer**	**Annealing Temperature**
NF-κb	5'-CAATGGCTACACAGGACCA-3'	5′-CACTGTCACCTGGAACCAGA-3′	52° C
TNF-α	5'-TCTACTGAACTTCGGGGTGATCG-3'	5'-TGATCTGAGTGTGAGGGTCTGGG-3'	56° C
p53	5'-ACCGCCGACCTATCCTTACC-3'	5'-TCTTCTGTACGGCGGTCTCTC-3'	56° C
β-actin	5'-ACGGCCAGGTCATCACTATTG-3'	5'-CAAGAAGGAAGGCTGGAAAAGA-3'	52° C
miRNA-29a	5′-GCGCACTGATTTCTTTTGGTGTTCAG–3'	5′-GCGAGCACAGAATTAATACGAC-3'	51° C
RNU6B	5′-CTCGCTTCGGCAGCACATA–3'	5′-CGCTTCACGAATTTGCGTG-3'	53° C
